# Predictors of mortality following emergency open colectomy for ischemic colitis: a single-center experience

**DOI:** 10.1186/s13017-020-00321-4

**Published:** 2020-06-29

**Authors:** Nassiba Beghdadi, Elisa Reitano, Frederic Cochennec, Pascal Desgranges, Aurelien Amiot, Iradj Sobhani, Nicolas Mongardon, Olivier Langeron, Margherita Notarnicola, Sébastien Mulé, Alain Luciani, Florence Canoui-Poitrine, Alexis Laurent, Daniele Sommacale, Francesco Brunetti, Nicola de’ Angelis

**Affiliations:** 1 Unit of Digestive and HPB surgery, CARE Department, Henri Mondor Hospital, AP-HP, and Université Paris Est, UPEC, 51 Avenue du Maréchal de Lattre de Tassigny, 94010 Créteil, France; 2 Unit of vascular surgery, CARE Department, Henri Mondor Hospital, AP-HP, and Université Paris Est, UPEC, Créteil, France; 3 Department of Gastroenterology and Digestive Endoscopy, Henri Mondor Hospital, AP-HP, and Université Paris Est, UPEC, Créteil, France; 4 Service d’anesthésie-réanimation chirurgicale, DMU CARE, DHU A-TVB, Assistance Publique-Hôpitaux de Paris (AP-HP), Hôpitaux Universitaires Henri Mondor, 94010 Créteil, France; 5 Université Paris Est Creteil, Faculté de Santé, 94010 Créteil, France; 6 Unit of Radiology, Henri Mondor Hospital, AP-HP, and Université Paris Est, UPEC, Créteil, France; 7 Inserm U955, Team 18, Créteil, France; 8 Clinical Epidemiology and Ageing Unit, Henri Mondor Hospital, APHP, EA 7376, CEpiA-IMRB, Université Paris Est, Créteil, France

**Keywords:** Ischemic colitis, Abdominal surgery, Emergency surgery, Aortic surgery

## Abstract

**Background:**

Ischemic colitis (IC) is a severe emergency in gastrointestinal surgery. The aim of the present study was to identify the predictors of postoperative mortality after emergent open colectomy for IC treatment. Additionally, we compared postoperative outcomes of patients undergoing emergent colectomy due to aortic surgery-related IC (AS-IC group) vs. other IC etiologies (Other-IC group).

**Methods:**

We analyzed records of consecutive patients who underwent emergency open colectomy for IC between 2008 and 2019. Logistic regression analysis was performed to identify clinical and operative parameters associated with postoperative mortality. The AS-IC and Other-IC groups were compared for mortality, morbidity, ICU stay, hospital stay, and survival.

**Results:**

During the study period, 94 patients (mean age, 67.4 ± 13.7 years) underwent emergent open colectomy for IC. In the majority of cases, IC involved the entire colon (53.2%) and vasopressor agents were required preoperatively (63.8%) and/or intraoperatively (78.8%). Thirty-four patients underwent surgery due to AS-IC, whereas 60 due to Other-IC causes. In the AS-IC group, 9 patients had undergone endovascular aortic repair and 25 open aortic surgery; 61.8% of patients needed aortic surgery for ruptured abdominal aortic aneurism (AAA). Overall, 66 patients (70.2%) died within 90 days from surgery. The AS-IC and Other-IC groups showed similar operative outcomes and postoperative complication rates. However, the duration of the ICU stay (19 days vs. 11 days; *p* = 0.003) and of the total hospital stay (22 days vs. 16 days; *p* = 0.016) was significantly longer for the AS-IC group than for the Other-IC group. The rate of intestinal continuity restoration at 1 year after surgery was higher for the Other-IC group than for the AS-IC group (58.8% vs. 22.2%; *p* = 0.05). In the multivariate model, preoperative increased lactate levels, a delay between signs/symptoms’ onset and surgery > 12 h, and the occurrence of postoperative acute kidney injury were statistically associated with postoperative mortality. Neither IC etiology (aortic surgery vs. other etiology) nor ruptured AAA was associated with postoperative mortality.

**Conclusion:**

Emergency open colectomy for IC is associated with high postoperative mortality, which appears to be unrelated to the IC etiology. Preoperative lactate levels, > 12-h delay to surgery, and postoperative acute kidney injury are independent predictors of postoperative mortality.

## Introduction

Ischemic colitis (IC) is a severe emergency in gastrointestinal surgery [1], being associated with mortality rates of 50–80% [2, 3]. IC represents the most common type of intestinal ischemia with an estimated incidence of 15.6–17.7/100,000 person-years [4]. Recognized risk factors for IC are arterial hypertension, coronary artery disease, peripheral vascular disease, atrial fibrillation, diabetes mellitus, chronic kidney diseases, and hemodialysis [5–8]. Once diagnosed, adverse prognostic factors for IC-related mortality include age > 50 years [5], hemodynamic instability [5], isolated right colon involvement [7, 9], history of hypertension, and chronic kidney diseases [7, 8].

The etiologies of IC are numerous, but they can be divided into two main categories: occlusive and non-occlusive diseases [4]. In both cases, IC is the result of blood supply insufficiency to the colon leading to various severities of ischemic lesions, from superficial mucosal lesions to full-thickness transmural necrosis [10]. The clinical presentation is related to the severity and extent of the ischemia lesions. The most frequent symptoms are abdominal pain, diarrhea, mild lower gastrointestinal bleeding, and elevated or persistent fever [4, 11, 12].

IC is considered as one of the most severe postoperative complications of aortic surgery, occurring in 2% of patients operated on in an elective setting and in 9% of patients operated on in emergency [13, 14]. IC occurring after aortic surgery has been reported to increase mortality by 50% [15]. Several studies investigated the risk factors of IC after abdominal aneurysm (AAA) repair and compared the occurrence of IC after endovascular aortic repair (EVAR) vs. open aneurysm repair [16–19]. Older age, female gender, ruptured aneurysm, emergency surgery, prolonged surgery, aortic clamping duration, and previous cardiovascular history were associated with an increased risk of IC [5, 15, 17, 20, 21]. On the contrary, EVAR surgery was found to be protective towards IC compared to open aortic surgery probably due to lower ischemia-reperfusion injury and peripheral pro-inflammatory cytokine release [16, 22, 23].

Depending on the severity and IC grade, two treatment strategies can be discussed: a conservative approach or a surgical treatment [24, 25]. IC patients who require surgical treatment are most of the time operated on in an emergency setting, which is associated with a higher risk of postoperative mortality than elective surgery [14, 26, 27].

The aim of the present study was to identify predictors of postoperative mortality in patients requiring emergency open colectomy for IC treatment. Additionally, we compared postoperative outcomes of patients undergoing emergency colectomy due to aortic surgery-related IC vs. other IC etiologies.

## Methods

We scrutinized records of consecutive patients who underwent emergency open colectomy for IC at the Henri Mondor University Hospital of Creteil (France) between January 2008 and February 2019. The study protocol was approved by the Institutional Review Board (IRB: 00011558) and was conducted in conformity to the principles declared to the National Commission for Data Protection and Liberties (CNIL: 2210699) and in accordance with the ethical principles described in the Declaration of Helsinki.

### Study population

To be included in the present analyses, IC patients should have been treated with an emergency open colectomy; IC diagnosis should have been confirmed preoperatively by computed tomography scan and colonoscopy and then by histology on the surgical specimen. At the contrast-enhancement computed tomography scan, IC was identified as large bowel wall thinning (“paper-thin-wall”), or any large bowel wall thickening and/or abnormal wall enhancement. Porto-mesenteric venous gas, pneumatosis, free intra-peritoneal air, and mesenteric arterial or venous thrombus may also be present [5, 13, 28, 29]. Colonoscopy findings were classified according to Favier’s classification [30].

According to the IC etiology, patients were divided into 2 groups: those with aorta surgery-related IC (AS-IC group) and those with IC of other etiologies (Other-IC group). IC in the AS-IC group should have occurred during the aorta surgery hospitalization. We collected baseline characteristics including demographics, comorbidities, IC etiologies and location, and the need of preoperative vasopressor agents (norepinephrine/epinephrine). We estimated the Ischemic Colitis Mortality Risk (ICMR) score as described by Reissfelder et al. [31]. Operative and postoperative variables were also retrieved and compared between the groups.

### Emergency open colectomy procedure

The extent of the colonic resection depended on the severity of IC, based on preoperative and intraoperative findings, varying from segmental to total colectomies. After a midline laparotomy, the peritoneal cavity was explored. All necrotic, thickened, or edematous colonic segments were resected. The colon was transected in a macroscopically healthy area identified by the operating surgeon. For a left colon lesion, a Hartmann resection or a left colectomy, including the resection of the rectosigmoid junction, was performed. For IC located on the right colon, a right colectomy with resection of the right flexure and a double-barreled ileo-colostomy were performed. For ischemic lesions involving the right colon up to the splenic flexure, a subtotal colectomy was performed. For pancolic ischemia, a total colectomy with terminal ileostomy was performed. After surgery, all patients were admitted to the intensive care unit (ICU).

### Study outcomes

The main study outcome was postoperative mortality, defined as in-hospital mortality or within 90 days from colonic resection. Secondary outcomes included intraoperative variables, postoperative morbidity, and ICU stay and hospital stay duration. The hospital stay duration was counted starting from the day of the emergency colectomy for IC. Acute kidney injury was defined according to the KDIGO classification [32]. Myocardial infarction was defined as isolated troponin elevation, electrocardiogram changes, and/or clinical evidence of heart attack. Respiratory complications were defined as postoperative occurrence of pneumonia or need for prolonged intubation (> 48 h). Severity of postoperative complications was categorized by using the Clavien–Dindo Classification [33]. Survivors were followed up; during the first year, restoration of intestinal continuity was performed if suitable.

### Statistical analysis

Statistics were performed with tee SPSS software (Statistical Package for Social Science, IBM SPSS Statistics, version 23 for Macintosh; IBM Corp., Armonk, NY, USA). Continuous variables are expressed as mean (SD) or median (range), as appropriate. Categorical variables are expressed as frequency (*n*, %). Student *t* test, Mann–Whitney *U* test, Fisher exact test, or chi-square test was used for group comparisons, as appropriate. Logistic regression analysis was performed to identify clinical and operative parameters associated with postoperative mortality including the model variables that reached a *p* value < 0.2 at the univariate analysis. Odds ratios (OR) and adjusted OR (AOR) are presented with 95% confidence interval (CI). A *p* value ≤ 0.05 was considered as statistically significant.

## Results

During the study period, 94 patients underwent emergency open colectomy for IC. The study population had a mean age of 67.4 ± 13.7 years, with 65.9% being women. In the majority of cases, IC involved the entire colon (53.2%), requiring a total colectomy with terminal ileostomy. The time interval from signs/symptoms onset to surgery was > 12 h in 75.5% of patients. The majority of patients needed vasopressors preoperatively (63.8%) and/or intraoperatively (78.8%) (Table 1).

**Table 1:** Summary of the clinical characteristics of patients undergoing emergent colectomy for ischemic colitis (*n* = 94)

Variables	Whole sample
Gender (F/M) [*n*]	62/32
Age, years [mean (SD)]	67.44 (13.70)
Age > 75 years [*n* (%)]	34 (36.2)
BMI (kg/m^2^) [mean (SD)]	23.25 (4.98)
BMI > 30 (kg/m^2^) [*n* (%)]	11 (11.7)
Preoperative leukocytes (10^9^/L) [mean (SD)]	14.51 (9.69)
Preoperative hemoglobin (g/L) [mean (SD)]	10.33 (2.71)
Preoperative creatinine (mg/dL) [mean (SD)]	195.55 (142.09)
Preoperative lactates (mg/dL) [mean (SD)]	5.4 (4.31)
Preoperative pH [mean (SD)]	7.25 (0.15)
Protrombin ratio (% of normal) [mean (SD)]	73 (15.36)
Diabetes [*n* (%)]	16 (17)
Severe coronary disease [*n* (%)]	70 (74.5)
Previous TIA/stroke [*n* (%)]	8 (8.5)
Hypertension [*n* (%)]	66 (70.2)
COPD [*n* (%)]	20 (21.3)
Smoking [*n* (%)]	30 (31.9)
Kidney failure [*n* (%)]	24 (25.5)
Previous carotid surgery [*n* (%)]	6 (6.4)
Previous aortic stenting [*n* (%)]	14 (14.9)
Previous legs vascular surgery [*n* (%)]	12 (12.8)
Previous colectomy [*n* (%)]	2 (2.1)
Comorbidity > 1 [*n* (%)]	42 (44.7)
Cardioasa therapy [*n* (%)]	19 (20.2)
Oral anticoagulant therapy [*n* (%)]	24 (25.5)
Charlson Index [mean (SD)]	5.41 (2.69)
ASA Score [*n* (%)]	
*II*	10 (10.6)
*III*	58 (61.7)
*IV*	26 (27.7)
Time interval from signs/symptoms onset to surgery [*n* (%)]	
*< = 12 h*	23 (24.5)
*> 12 h*	71 (75.5)
Localization of ischemia [*n* (%)]	
*Left colon*	19 (20.2)
*Right colon*	4 (4.3)
*Transverse*	6 (6.4)
*Right and transverse*	15 (16)
*Entire colon*	50 (53.2)
Etiology of ischemia [*n* (%)]	
*Aortic surgery*	34 (36.2)
*Open surgery*	25
*EVAR*	9
*Other etiologies*	60 (63.8)
Presence of colic perforation [*n* (%)]	20 (21.3)
Presence of aneurysmatic rupture [*n* (%)]^a^	21 (61.8)
Type of colonic resection [*n* (%)]	
*Hartman’s resection*	19 (20.2)
*Subtotal colectomy with ileo-sigmoid colostomy*	21 (22.3)
*Total colectomy with terminal ileostomy*	50 (53.2)
*Right colectomy with ileo-colonostomy*	4 (4.3)
Open abdomen needed [*n* (%)]	4 (4.3)
Preoperative vasopressor agents [*n* (%)]	60 (63.8)
Intraoperative vasopressor agents [*n* (%)]	74 (78.8)

*ASA* American Society of Anesthesiologist classification, *BMI* body mass index, *COPD* chronic obstructive pulmonary disease, *IC* ischemic colitis *TIA* transient ischemic attack

^a^Regarding patients with AS etiology

Concerning the cause of IC, 34 patients (36.2%) presented with an AS-IC and 60 patients (63.8%) with an Other-IC. In the AS-IC group, 9 patients had undergone EVAR, and 25 open aortic surgery; overall, 21/34 patients (61.8%) needed aortic surgery for AAA rupture. The Other-IC causes included occlusive diseases (e.g., trauma (4), arterial emboli (4), radiation (3), thrombosis emboli (2)) and non-occlusive diseases (e.g., colon cancer (2), cocaine abuse (1); *Clostridium difficile* (2), inflammatory bowel disease (3), non-steroidal anti-inflammatory drugs (5), volvulus (5), hemodialysis (5), hypo-perfusion (10)). The median follow-up period was 36 months (range, 9–60 months).

Operative and postoperative outcomes for the entire study populations are summarized in Table 2. Overall, 66 patients (70.2%) died within 90 days from surgery.

**Table 2:** Summary of operative and postoperative characteristics of patients undergoing emergent colectomy for ischemic colitis

Variables	Whole sample
Operative time, min [mean (SD)]	151.54 (55.33)
Estimated blood loss, mL [median (range)]	450 (100–890)
Transfused patients [*n* (%)]	50 (53.2)
Postoperative multi-organ failure [*n* (%)]	56 (59.6)
Postoperative acute kidney injury [*n* (%)]	51 (54.3)
Postoperative liver failure [*n* (%)]	5 (5.3)
Postoperative heart attack [*n* (%)]	8 (8.5)
Postoperative stroke [*n* (%)]	2 (2.1)
Postoperative metabolic acidosis [*n* (%)]	1 (1.1)
Postoperative respiratory failure [*n* (%)]	17 (18.1)
Postoperative low output syndrome [*n* (%)]	5 (5.3)
Postoperative sepsis [*n* (%)]	38 (40.4)
Postoperative complication severity (Clavien–Dindo Classification) [*n* (%)]	
III/IV	28 (29.8)
V	66 (70.2)
Patients in ICU [*n* (%)]	94 (100)
Duration of ICU stay, days [mean (SD)]	14.50 (17.17)
Reoperation during the same admission [*n* (%)]	18 (19.1)
Hospital stay, days [mean (SD)]	18.26 (20.85)
Postoperative mortality at 90 days [*n* (%)]	66 (70.2)
Survival at 1 year [*n* (%)]	26 (27.6)
Disposition* [*n* (%)]	
*Home*	11 (39.3)
*Rehabilitation hospital/elderly house*	17 (60.7)
Restoration of intestinal continuity at 1 year after surgery** [*n* (%)]	12 (46.1)
Mortality after restoration of intestinal continuity at 1 year after surgery [*n* (%)]	0
Survival status at the last follow-up visit*	
*Alive*	22 (75.9)
*Dead for IC-related causes*	3 (10.3)
*Dead for other causes*	3 (10.3)

*ICU* intensive care unit

*n = 28

**n = 26

### Comparison of AS-IC vs. Other-IC patients

The clinical characteristics of patients in the AS-IC vs. Other-IC group are presented in Table 3. Significant between-group differences were found only for the prevalence of hypertension, the location of ischemia and consequently the extent of colonic resection, and the use of open abdomen. Comparisons between the AS-IC group and Other-IC group for postoperative outcomes are presented in Table 4. After adjustment on the demographic and clinical variables that were significantly different between the AS-IC and Other-IC groups, the two groups showed similar rates of postoperative multi-organ failure, acute kidney injury, respiratory failure, and postoperative mortality (Table 4). However, the ICU stay (19 days vs. 11 days; *p* = 0.003) and the total hospital stay durations (22 days vs. 16 days; *p* = 0.016) were significantly longer for the AS-IC group than for the Other-IC group. The rate of restoration of the intestinal continuity at 1 year was higher in the Other-IC group than in the AS-IC group (58.8% vs. 22.2%; *p* = 0.05) (Table 4).

**Table 3:** Summary of the clinical characteristics of IC patients undergoing emergency open colectomy due to aorta surgery-related IC (AS-IC group) vs. other etiologies (Other-IC group)

Variables	AS-IC group (*n* = 34)	Other-IC group (*n* = 60)	*p* value
Gender (F/M) [*n*]	9/25	23/37	0.267
Age, years [mean (SD)]	69.74 (10.73)	66.13 (15.05)	0.223
Age > 75 years [*n* (%)]	14 (41.2)	20 (33.3)	0.506
BMI (kg/m^2^) [mean (SD)]	22.8 (4.92)	23.46 (5.05)	0.634
BMI > 30 (kg/m^2^) [*n* (%)]	4 (11.8)	7 (11.7)	1
Preoperative leukocytes (10^9^/L) [mean (SD)]	11.94 (8.18)	15.90 (10.22)	0.062
Preoperative hemoglobin (g/L) [mean (SD)]	9.81 (2.25)	10.64 (2.93)	0.162
Preoperative creatinine (mg/dL) [mean (SD)]	182.29 (109.72)	201.78 (155.62)	0.583
Preoperative lactates (mg/dL) [mean (SD)]	5.57 (4.19)	5.31 (4.42)	0.796
Preoperative pH [mean (SD)]	7.27 (0.16)	7.25 (0.15)	0.524
Protrombin ratio (% of normal) [mean (SD)]	73 (15.36)	71.59 (10.58)	0.520
Diabetes [*n* (%)]	6 (17.6)	10 (16.7)	1
Severe coronary disease [*n* (%)]	26 (76.5)	44 (73.3)	0.809
Previous TIA/stroke [*n* (%)]	1 (2.9)	7 (11.7)	0.251
Hypertension [*n* (%)]	29 (85.3)	37 (61.7)	**0.019**
COPD [*n* (%)]	10 (29.4)	10 (16.7)	0.191
Smoking [*n* (%)]	15 (44.1)	15 (25)	0.068
Kidney failure [*n* (%)]	9 (26.5)	15 (25)	1
Previous carotid surgery [*n* (%)]	1 (2.9)	5 (8.3)	0.413
Previous aortic stenting [*n* (%)]	8 (23.5)	6 (10)	0.129
Previous legs vascular surgery [*n* (%)]	4 (11.8)	8 (13.3)	1
Previous colectomy [*n* (%)]	1 (2.9)	1 (1.7)	1
Comorbidity > 1 [*n* (%)]	16 (47.1)	26 (43.3)	0.830
Cardioasa therapy [*n* (%)]	5 (14.7)	14 (23.3)	0.426
Oral anticoagulant therapy [*n* (%)]	11 (32.4)	13 (21.7)	0.326
Charlson Index [mean (SD)]	5.47 (2.29)	5.37 (2.91)	0.867
ASA score [*n* (%)]			0.129
*II*	1 (2.9)	9 (15)	
*III*	21 (61.8)	37 (61.7)	
*IV*	12 (35.3)	14 (23.3)	
Time interval from signs/symptoms onset to surgery [*n* (%)]			0.467
*< = 12 h*	9 (26.5)	14 (23.3)	
*> 12 h*	25 (73.5)	46 (76.7)	
Location of ischemia [*n* (%)]			**0.015**
*Left colon*	9 (26.4)	10 (16.6)	
*Right colon*	4 (11.8)	0 (0)	
*Transverse*	3 (8.8)	3 (5)	
*Right and transverse*	2 (5.9)	13 (21.7)	
*Entire colon*	16 (47.1)	34 (56.7)	
Presence of colic perforation [*n* (%)]	6 (17.6)	14 (23.3)	0.426
Type of colonic resection [*n* (%)]			**0.020**
*Hartman’s resection*	9 (26.5)	10 (16.7)	
*Subtotal colectomy with ileo-sigmoid colostomy*	5 (14.7)	16 (26.7)	
*Total colectomy with terminal ileostomy*	16 (47.1)	34 (56.6)	
*Right colectomy with ileo-colonostomy*	4 (11.7)	0	
Open abdomen needed [*n* (%)]	4 (11.8)	0	**0.015**
Preoperative vasopressor agents [*n* (%)]	19 (55.9)	41 (68.3)	0.267
Intraoperative vasopressor agents [*n* (%)]	27 (79.4)	47 (78.3)	0.815

*AS* aortic surgery, *ASA* American Society of Anesthesiologist classification, *BMI* body mass index, *COPD* chronic obstructive pulmonary disease, *IC* ischemic colitis, *TIA* transient ischemic attack

**Table 4:** Operative and postoperative outcomes of IC patients undergoing emergency open colectomy due to aorta surgery-related IC (AS-IC group) vs. other etiologies (Other-IC group)

Variables	AS-IC group (*n* = 34)	Other-IC group (*n* = 60)	*p* value	Adjusted *p* value^#^
Operative time, min [mean (SD)]	147.67 (47.68)	153.82 (59.71)	0.632	0.754
Estimated blood loss, mL [median (range)]	450 (100–890)	460 (40–1000)	0.178	0.102
Transfused patients [*n* (%)]	19 (55.9)	31 (51.7)	0.830	0.352
Postoperative MOF [*n* (%)]	19 (55.9)	37 (61.7)	0.664	0.292
Postoperative kidney failure [*n* (%)]	20 (58.1)	31 (51.7)	0.526	0.382
Postoperative liver failure [*n* (%)]	1 (2.9)	4 (6.7)	0.650	0.998
Postoperative heart attack [*n* (%)]	4 (11.8)	4 (6.7)	0.454	0.358
Postoperative metabolic acidosis [*n* (%)]	0	1 (1.7)	1	0.997
Postoperative respiratory failure [*n* (%)]	7 (20.6)	7 (11.7)	0.366	0.419
Postoperative low output syndrome [*n* (%)]	1 (2.9)	4 (6.7)	0.650	0.440
Postoperative complications (Clavien–Dindo Classification) [*n* (%)]			1	0.307
III/IV	10 (29.4)	18 (30)		
V	24 (70.6)	42 (70)		
Patients in ICU [*n* (%)]	34 (100)	60 (100)	1	NA
Duration of ICU stay, days [mean (SD)]	19.06 (21.23)	11.92 (13.93)	0.085	**0.003 [adjusted mean difference 12.09 (3.98)]**
Hospital stay, days [mean (SD)]	22.20 (23.31)	16.03 (19.16)	0.169	**0.016 [adjusted mean difference 12.14 (4.96)]**
Postoperative mortality at 90 days [*n* (%)]	24 (70.6)	42 (70)	1	0.428
Survival at 1 year [*n* (%)]	9 (90)	17 (89.5)	1	0.999
Disposition* [*n* (%)]			0.895	0.790
*Home*	4 (40)	7 (38.9)		
*Rehabilitation hospital/elderly house*	6 (60)	11 (61.1)		
Restoration of intestinal continuity at 1 year after surgery** [*n* (%)]	2 (22.2)	10 (58.8)	0.110	**0.05**
Mortality after restoration of intestinal continuity at 1 year after surgery [*n* (%)]	0	0	NA	NA

*MOF* multi-organ failure, *ICU* intensive care unit

*n = 28

**n = 26

^*#*^Model adjusted on demographic and clinical variables significantly different between AS-IC and Other-IC groups: hypertension, localization of ischemia, type of colonic resection, and use of open abdomen

### Predictors of postoperative mortality

At the univariate analysis, lactate level > 2.5 mmol/L, time from signs/symptoms’ onset to surgery > 12 h, need of preoperative and/or intraoperative adrenergic vasopressors, sepsis, and blood loss > 500 mL were found to be significantly associated with postoperative mortality (Table 5). In the multivariate model, preoperative lactate levels, time from signs/symptoms onset to surgery, and postoperative acute kidney injury were statistically associated with the occurrence of postoperative mortality. The presence of AAA rupture and the IC etiology (aortic surgery vs. other etiology) were not significantly associated with postoperative mortality (Table 5).

**Table 5:** Factors associated with postoperative mortality

Variables	Univariate analysis			Multivariate analysis	
	In-hospital postoperative mortality	Odds ratio (95%CI)	*p* value	Adjusted odds ratio (95%CI)	*p* value
Age > 75 years [*n* (%)]	27 (79.4)	2.23 (0.83–5.96)	0.163		
Age > 80 years [*n* (%)]	14 (77.8)	1.71 (0.51–5.75)	0.571		
Age, years (per unit increase)	NA	1.02 (0.99–1.06)	0.098	1.03 (0.98–1.09)	0.157
Female [*n* (%)]	44 (71)	1.28 (0.51–3.18)	0.641		
ASA score > III [*n* (%)]	21 (80.8)	2.29 (0.76–6.84)	0.211		
Pancolic ischemia [*n* (%)]	35 (70)	1.08 (0.45–2.61)	1		
Obesity [*n (*%)]	7 (63.6)	1.32 (0.35–4.92)	0.733		
BMI (per unit increase)	NA	1.01 (0.92–1.12)	0.696		
Hypoalbuminemia (< 3.5 g/dL) [*n* (%)]	4 (80)	1.83 (0.19–17.18)	1		
Lactates > 2.5 mmol/L [*n* (%)]	**56 (76.7)**	**4.39 (1.58–12.18)**	**0.006**	**11.85 (1.64**–**85.17)**	**0.014**
Preoperative hemoglobin level (per unit increase)	NA	1 (0.83–1.20)	0.973		
Diabetes [*n* (%)]	56 (71.8)	1.98 (0.65–5.97)	0.244		
Severe coronary disease [*n* (%)]	50 (71.4)	1.5 (0.56–3.97)	0.449		
COPD [*n* (%)]	50 (67.6)	1.44 (0.47–4.54)	0.596		
Kidney failure [*n* (%)]	47 (67.1)	1.47 (0.51–4.34)	0.611		
Hypertension [*n* (%)]	17 (60.7)	1.72 (0.68–4.38)	0.329		
Smoking [*n* (%)]	43 (67.2)	1.35 (0.51–3.57)	0.636		
Comorbidity > 1 [*n* (%)]	33 (78.6)	2.29 (0.90–5.78)	0.115	1.81 (0.33–9.9)	0.490
Time from signs/symptoms onset to surgery > 12 h [*n* (%)]	**60 (84.5)**	**19.63 (6.02–63.97)**	**< 0.0001**	**34.99 (7.14**–**171.33)**	**< 0.0001**
Presence of aneurysmatic rupture* [*n* (%)]	8 (61.5)	2 (0.44–9)	0.451		
EVAR surgery* [*n* (%)]	4 (44.4)	0.2 (0.038–1.03)	0.085		
AS [*n* (%)]	24 (70.6)	1.11 (0.44–2.78)	1		
Total colectomy [*n* (%)]	35 (70)	1.08 (0.45–2.61)	1		
Preoperative vasopressor agents [*n* (%)]	**47 (78.3)**	**3.21 (1.29–8)**	**0.019**	2.54 (0.20–31.12)	0.466
Intraoperative vasopressor agents [*n* (%)]	**55 (74.3)**	**2.89 (1.04–8)**	**0.037**	6.37 (0.87–46.6)	0.068
Sepsis [*n* (%)]	**32 (84.2)**	**3.71 (1.33–10.32)**	**0.012**	2.31 (0.61–8.79)	0.218
Low output syndrome [*n* (%)]	5 (100)	1.48 (1.28–1.71)	0.125	3.83 (0.3–48.97)	0.301
Postoperative acute kidney injury [*n* (%)]	39 (76.5)	2.12 (0.87–5.17)	0.118	**5.09 (1.13**–**22.8)**	**0.033**
Operative time, min (per unit increase)	NA	1 (0.99–1.01)	0.493		
Blood loss > 500 mL [*n* (%)]	**45 (78.9)**	**3.18 (1.28–7.89)**	**0.013**	2.48 (0.68–9.05)	0.169
Open abdomen needed [*n* (%)]	4 (100)	1.47 (1.27–1.7)	0.172	0 (0–0)	0.999
ICMR score (per unit increase)	NA	1.37 (0.89–2.12)	0.146	2.85 (0.91–8.91)	0.072

*ASA* American Society of Anesthesiologists, *AS* for aortic surgery, *COPD* for chronic obstructive pulmonary disease, *EVAR* endovascular aortic repair, *ICMR* Ischemic Colitis Mortality Risk

*n = 34

## Discussion

The present single-center study showed that IC requiring an emergency open colectomy is associated with a high postoperative mortality, which can be predicted by high levels of preoperative lactates, delayed interval from signs/symptoms onset to surgery (> 12 h), and the occurrence of postoperative acute kidney injury. On the contrary, the specific etiology of IC does not impact on the outcome.

Prediction of postoperative outcomes and risk assessment can be of particular importance facing a pathology that requires an optimized emergency management. In this perspective, some authors proposed different predictive scores aiming at guiding the clinical treatment choice and anticipating postoperative complications after colonic resection for IC [5, 34, 35].

In 2010, Chung et al. [35] proposed a prognostic model able to accurately identify severe IC requiring surgery or life-threatening based on 3 preoperative variables: tachycardia, shock within 24 h after admission, and endoscopic evidence of ulceration. The probability of severe IC was 1% if none of these 3 variables was present and was 74% if the 3 components were all present [35]. This score is of interest to assess the patient’s risk once IC is diagnosed and prior to surgery. By providing a method to assess a patient’s prognosis, it guides clinicians in the choice of the best management strategy and postoperative protocol, which must take into account the individual patient’s risk.

In 2011, Reissfelder et al. [31] developed the ICMR score, a risk score to predict perioperative mortality in patients undergoing surgery for IC. In 2013, Castleberry et al. [34] assessed the validity of the ICMR score in their 10-year cohort composed of 115 patients who underwent colectomy for acute IC. They reported an adequate discriminatory accuracy in predicting in-hospital mortality with an area under the receiver operating characteristic curve of 0.75. When analyzed separately, the individual components of the ICMR score that performed better were the preoperative lactate levels > 2.5 mmol/L, the occurrence of postoperative kidney injury requiring hemodialysis, and the use of intraoperative vasopressor. On the contrary, the non-occlusive etiology of IC and the need of total/subtotal colectomy were not significantly associated with in-hospital mortality [34].

In accordance with the previous literature (Table 6), the present study confirms the predictive value of the preoperative lactate levels and the occurrence of postoperative acute kidney injury, whereas the need of intraoperatory adrenergic vasopressors and the ICRM score showed only a statistical trend. We also observed that the delay to surgery can significantly impact on the risk of in-hospital mortality, being those operated on after > 12 h from signs/symptom onset drastically more at risk than those receiving an early diagnosis and a prompt surgical treatment.

**Table 6:** Summary of the most relevant studies in the literature on IC-related mortality following emergency colectomy

Author, year	Study design	Study time frame	Number	Mean or median age (years)	IC etiology	IC localization	IC requiring surgery (by procedure)	IC mortality	Risk factors for IC mortality	Survival
**Aday et al., 2018**	Retrospective single-center study	2012–2016	275	62	Cardiovascular surgery	NA	Overall, 5% EVAR, 28.5% open surgery, 71.5%	nrAAA, 62.5% rAAA, 83%	NA	NA
**Gilshtein et al., 2018**	Retrospective single-center study	2011–2016	63	72.5	NA	NA	Overall, 19%	50%	- Older age - Comorbidities (chronic renal disease, ischemic heart disease) - Higher lactate level	NA
**Noh et al., 2015**	Retrospective single-center study	2003–2011	50	68	Medical causes	Right colon, 22% Left colon, 66% Transverse colon, 6% Pancolic, 6%	100%	30%	- History of cardiovascular surgery - Delay to surgery ≥ 3 days	NA
**Genstorfer et al., 2014**	Retrospective single-center study	2004–2010	100	74	Aortic surgery, 16% Cardiac surgery, 17%	Right colon, 33% Left colon, 40% Pancolic, 27%	100%	54%	- Right colon ischemia - Pancolic ischemia - Decrease preoperative pH - Prior cardiac/aortic surgery	NA
**Moszkowicz et al., 2014**	Retrospective single-center study	1997–2012	191	70	Aortic surgery, 62% Spontaneous, 38%	AS-IC: Right colon, 2% Left colon, 45% Pancolic, 53% Spontaneous IC: Right colon, 60% Left colon, 13% Pancolic, 6%	91%	48%	- Age older than 75 years - Multiple organ failure	NA
**Castleberry et al., 2013**	Retrospective single-center study	2000–2009	115	64	GI surgery, 12% Cardiovascular surgery, 26%	Right colon, 49% Pancolic, 26%	100%	In hospital, 37%	- ASA > 4 - Preoperative lactate level - Renal failure requiring hemodialysis - Intraoperative adrenergic vasopressor use - Blood loss > 500 mL	1 year, 43% 3 years, 33% 5 years, 27%
**Reissfelder et al., 2011**	Retrospective single-center study	2002–2008	177	69	NA	NA	100%	48%	- Non-occlusive IC - Acute renal failure - Extent of bowel ischemia - Lactate level - Duration of catecholamine therapy	NA
**Antolovic et al., 2008**	Retrospective single-center study	2001–2004	85	68.5	Cardiovascular surgery, 55%	Right colon, 26% Left colon, 8% Sigmoid, 5% Pancolic, 49%	100%	47%	- ASA >3 - Emergency surgery - Blood loss	NA
**Huguier et al., 2006**	Retrospective single-center study	1992–1999	73	73	NA	Pancolic, 9%	45%	62%	- Age < 80 years - Male gender - Absence of bleeding - Abdominal tenderness	2 years, 88% 5 years, 68%
**Present study**	Retrospective single-center study	2008–2019	94	72	Aortic surgery, 36.2% Other causes, 63.8%	Right colon, 4.3% Left colon, 20.2% Transverse colon, 6.4% Pancolic, 53.2%	Overall, 100% EVAR, 26.% Open surgery, 73.5% rAAA, 61.8%	In hospital/90 days, 70.2%	- Preoperative lactates level - Time interval from symptoms onset to surgery - Intraoperatory adrenergic vasopressors - Postoperative kidney failure - ICMR score	1 year, 27.6%

*IC* ischemic colitis, *AAA* aortic abdominal repair, AFib/RVR Atrial fibrillation with rapid ventricular response, *EVAR* endovascular aortic repair, *GI* gastrointestinal, *IMA* inferior mesenteric artery, *nrAAA* non-ruptured AAA, *rAAA* ruptured AAA, *RCI* right colonic ischemia, *SMA* superior mesenteric artery

The definition of immediate and delayed surgery varies among the studies [8, 20, 31, 36, 37]. In some studies, as in the present one, immediate surgery was defined as the surgical treatment performed within 12 h from admission or surgical consultation, whereas delayed surgery as the intervention performed more than 12 h after admission owing to severe or worsening clinical conditions, or no response to conservative therapy [8]. As known, the clinical presentation of IC can be vague and unspecific, and the frequent presence of pre-existing medical conditions may hamper a timely diagnosis [4, 26, 38]. Severe cases at presentation have more chance to be rapidly taken in the operating room, but they are also associated with poorer outcomes due to the severity and extent of the ischemic lesion [4, 5, 8, 15, 26, 31, 36, 39]. When comparing mortality rates after emergency colectomy for IC, it is thus important to take into account the type of procedure performed (e.g., segmental resection, total colectomy), the surgical setting (e.g., emergency or delayed surgery), and the patient’s status (e.g., comorbidities, hemodynamic stability). Some authors advocated for an aggressive approach with extended colonic resections (e.g., left colectomy or subtotal colectomy) and only rarely limited to a segment (e.g., right colectomy) [3, 10, 13]. Indeed, acute IC located in the right colon should be considered as an extended colonic hypo-perfusion with global hypo-perfusion in the mesenteric superior artery territory. However, an aggressive management is not supported by all surgeons. Paterno et al. [8] suggested that in case of limited IC extension and no hemodynamic instability, a segmental colon resection is an effective option, without increased mortality. In the present study, we considered a sample of IC patients all requiring emergency surgery; in 53.2% of cases, IC involved the entire colon. The colonic resection was adapted to the IC extension, leading to a great majority of subtotal or total colectomies.

Previous studies reported high mortality rates for IC developed after ruptured AAA repair surgery [15, 16, 20, 26, 40], with some differences related to the type of aortic surgery [14, 22, 40]. Indeed, a recent systematic review and meta-analysis demonstrated that IC occurs more frequently after open repair (2.1–3.6%) than in EVAR (0.5–1%) in the elective setting [22]. However, once IC occurred and an emergency colectomy is required, the mortality appears to be independent of the type of aortic surgery performed. The present study confirms these findings and suggests that there is no evidence of an impact on mortality of the specific IC etiology, namely the aortic surgery-related cause or other causes, including occlusive and non-occlusive diseases.

In the present study, survivors of open colectomy underwent a restoration of the intestinal continuity in almost 50% of cases, with no related postoperative mortality. Interestingly, the rate of intestinal continuity restoration within 1 year from surgery was higher for patients in the Other-IC group than in the AS-IC one. This difference may be explained by several factors, such as the severity of comorbid diseases in AS-IC patients. Indeed, these patients showed most of the time cardiovascular comorbidities, which have detrimental effects on disease evolution and may be related to worse postoperative outcomes. In the study by Castleberry et al. [34], 24% of IC survivors underwent elective end-ostomy reversal with a mortality rate of 18%. Mariani et al. [41] described good outcomes after the restoration of intestinal continuity following colectomy for IC. This was possible in 40% of survivors, and the median delay between colectomy and the intestinal continuity restoration was 7.9 months (range 0.2–35 months). There was no postoperative mortality, but 13% developed surgical complications (e.g., lower digestive tract bleeding, wound infection) and 33% medical complications (e.g., pneumonia, cardiac failure, acute renal failure) [41]. The authors did not find any clinical or biological discriminant factor that may help in identifying which patients should undergo reversal surgery. However, they supported that the restorati0on of intestinal continuity should be discussed in multidisciplinary meeting for every surviving IC patient, and although challenging, it should be considered to improve quality of life in selected, motivated patients [41]. In the same vein, further studies may explore the possibility to manage IC by emergency laparoscopy to obtain additional advantages in the postoperative period related to the minimally invasive approach [42, 43]. However, this may be conceivable only in highly selected patients, depending on the IC severity and etiology (non-related to aorta surgery).

The present study has limitations that must be considered when interpreting the results. It is a retrospective analysis of an 11-year cohort of IC patients requiring emergency surgery. Patients were treated in a single institution over a long period of time; although the health database was prospectively maintained, reporting and selection bias cannot be ruled out. Furthermore, the external validity of the findings is limited. However, the present study investigated a relatively large sample of IC patients all treated by open colectomy in an emergency setting. Studies in the literature are rather heterogeneous in methods and target populations, making direct comparisons difficult. Efforts should be made in future studies, both prospective and retrospective, to standardize outcome measures and preoperative evaluation, in order to facilitate study comparisons as well as a comprehensive appraisal of the body of literature.

## Conclusions

Emergency open colectomy for IC is associated with high postoperative mortality. The levels of preoperative lactates, a delay to surgery > 12 h, and the occurrence of postoperative acute kidney injury are found to be independent predictors of postoperative mortality. The specific etiology of IC, namely aortic surgery complications or other causes, appears to have no impact on postoperative mortality. These results support the key role of prompt diagnosis and surgical intervention in the management of severe IC. Moreover, the present data indicate that survivors in the Other-IC group are more likely to undergo intestinal continuity restoration within 1 year from IC surgery compared to AS-IC patients, which may have a considerable impact on IC survivors’ quality of life.

## Data Availability

The authors are responsible for the data described in the manuscript and assure full availability of the study material.

## Abbreviations

AAA: Abdominal aortic aneurism
AS: Aortic surgery
ASA: American Society of Anesthesiologist classification
BMI: Body mass index
COPD: Chronic obstructive pulmonary disease
EVAR: Endovascular aneurysm repair
IC: Ischemic colitis
ICU: Intensive care unit
MOF: Multi-organ failure
